# Preface for SEANUTS II

**DOI:** 10.1017/S1368980024002106

**Published:** 2025-02-13

**Authors:** Mary FF Chong

**Affiliations:** Saw Swee Hock School of Public Health, National University of Singapore and National University Health System, 117549 Singapore, Singapore

The triple burden of malnutrition has emerged as a critical public health issue, particularly in low- to middle-income countries. This phenomenon involves the coexistence of stunting, underweight, overweight, obesity and micronutrient deficiencies, impacting both children and adults. To address the long-term effects of malnutrition in children, comprehensive research is essential.

South East Asian Nutrition Surveys (SEANUTS) is a multi-centre initiative that assesses the nutritional status and dietary patterns of children aged 6 months to 12 years. The first SEANUTS conducted in four countries, namely Indonesia, Malaysia, Thailand and Vietnam, between 2009 and 2010, provided valuable evidence of the dual burden of malnutrition in Southeast Asia and influenced national planning of policies and programmes in the respective countries.

A decade on, the present SEANUTS II (2019–2021) builds on earlier foundation and aims to provide an updated and more comprehensive picture of the nutritional status of children aged 6 months to 12 years in the same four Southeast Asian countries, namely Indonesia, Malaysia, Thailand and Vietnam. SEANUTS II delves deeper and adds on further extensive measures including anthropometric measures, body composition assessments, dietary intake analysis, physical activity evaluations and blood biochemistry tests to determine micronutrient status.

Following standardised protocols across all sites to ensure data sharing and comparability, the success of SEANUTS II reflects the remarkable public–private partnerships among the communities (including schools and community health centers), academics from local and renowned universities and research institutes, government sectors and FrieslandCampina, the industry partner that sponsored and commissioned the study. The value of these concerted efforts and collaboration will be witnessed as findings from this study provide the evidence base for upcoming public health programmes and policies, which will need to be designed and tailored for specific contexts (e.g. urban *v*. rural, different age groups) in order to address both ends of the malnutrition spectrum simultaneously.

The current publications from the various countries under SEANUTS II underscore the need for regular monitoring of child nutritional status and, indeed, SEANUTS has set a precedent for this ongoing surveillance Importantly, findings from SEANUTS II will provide actionable insights for policymakers, healthcare professionals, researchers and communities to tackle the multi-faceted drivers of malnutrition and thereby continue to improve the health, development and well-being of children in Southeast Asia.

Guest Editor PHN Supplement SEANUTS II

